# Acupuncture Point Stimulation Treatments Combined With Conventional Treatment in Chronic Obstructive Pulmonary Disease: A Systematic Review and Network Meta-Analysis

**DOI:** 10.3389/fmed.2021.586900

**Published:** 2021-06-04

**Authors:** Cheng-Lin Tsai, Chou-Chin Lan, Chih-Wei Wu, Yun-Chun Wu, Chan-Yen Kuo, I-Shiang Tzeng, Pei-Shan Hsu, Chang-Ti Lee, Po-Chun Hsieh

**Affiliations:** ^1^Department of Chinese Medicine, Taipei Tzu Chi Hospital, Buddhist Tzu Chi Medical Foundation, New Taipei City, Taiwan; ^2^Division of Pulmonary Medicine, Taipei Tzu Chi Hospital, Buddhist Tzu Chi Medical Foundation, New Taipei City, Taiwan; ^3^School of Medicine, Tzu-Chi University, Hualien, Taiwan; ^4^College of Public Health, Institute of Epidemiology and Preventive Medicine, National Taiwan University, Taipei, Taiwan; ^5^Department of Research, Taipei Tzu Chi Hospital, Buddhist Tzu Chi Medical Foundation, New Taipei City, Taiwan

**Keywords:** acupressure massage, acupuncture, chronic obstructive pulmonary disease, moxibustion, health-related quality of life, FEV1% predicted, network meta-analysis

## Abstract

**Background:** Chronic obstructive pulmonary disease (COPD), which is a disease characterized by dyspnea, cough, and respiratory symptoms, leading to impaired health-related quality of life (HRQL) and exercise capacity, is highly prevalent worldwide. Some studies demonstrated that acupuncture point stimulation treatments (APSTs) are effective and safe in treating patients with COPD. The aim of this systematic review and network meta-analysis is to analyze the effects on HRQL and FEV1% predicted of diverse APSTs in treating patients with COPD.

**Materials and Methods:** We searched seven electronic databases. Randomized controlled trials (RCTs) with stable COPD patients comparing APSTs and conventional treatment (Tx) were included. The primary outcome was HRQL measured by COPD Assessment Test or St. George's Respiratory Questionnaire. The secondary outcome was FEV1% predicted. We performed random effect network meta-analysis using a consistency model.

**Results:** This network meta-analysis analyzed 21 RCTs with 1,577 stable COPD participants. In comparison with Tx, acupressure massage (AM) + Tx [−5.11; 95% confidence interval (CI), −6.65 to −3.57] was the most effective intervention in improving HRQL, followed by moxibustion (Mx) + Tx (−2.86; 95% CI, −3.86 to −1.86). Moreover, in comparison with Tx, Mx + Tx (7.79; 95% CI, 2.16 to 13.42) was the most effective intervention in improving FEV1% predicted, followed by acupuncture (A) + Tx (5.79; 95% CI, 2.90 to 8.68).

**Conclusions:** Combined interventions (APSTs + Tx) are more effective than single intervention in improving both HRQL and FEV1% predicted. AM, Mx, and A can be considered effective non-pharmacological complementary interventions in treating patients with COPD under Tx.

## Introduction

Chronic obstructive pulmonary disease (COPD) is one of the leading causes of disease and death worldwide (1). COPD is a disease characterized by airway obstruction combined with systemic and airway inflammation (2). Patients diagnosed with COPD experienced chronic cough with sputum production, progressive dyspnea, acute exacerbation, and more severe symptoms such as anorexia (3). COPD reduces patients' health-related quality of life (HRQL) and increases the disability-adjusted of life years (4). Comprehensive managements including pharmacological and non-pharmacological treatments are suggested by the Global Initiative for Chronic Obstructive Lung Disease (GOLD) guidelines and are proven to be beneficial for patients with COPD with releasing symptoms, reducing the frequency of acute exacerbation (3). However, several patients with COPD still experience dyspnea and poor HRQL despite undergoing several treatments suggested by the GOLD guidelines (1). Therefore, determining alternative treatments to treat COPD is required.

Acupuncture points, which are defined as specific points on the body surface, are applied to various acupuncture point stimulation treatments (APSTs), such as acupuncture, warm acupuncture, electroacupuncture, acupoint bloodletting, acupoint embedding, moxibustion (Mx), acupoint sticking therapy, acupressure massage (AM), acu-transcutaneous electrical nerve stimulation (Acu-TENS), and laser acupuncture. Our previous meta-analysis indicated that body acupuncture therapy improves HRQL of patients with stable COPD even under conventional treatments (Tx) (5). Suzuki et al. reported that acupuncture can be a useful adjunctive therapy to improve the nutritional status of patients with COPD (6). Liu et al. indicated that Acu-TENS over acupuncture points improved FEV1% predicted and COPD Assessment Test (CAT) scores in patients with stable COPD (7). AM combined with Tx improved CAT scores in patients with stable COPD (8). Mx combined with rehabilitation training improved St. George's Respiratory Questionnaires (SGRQ) scores (9). However, a study investigating the effectiveness between APSTs has not been conducted yet. Hence, a comparison between different APSTs is necessary.

Network meta-analysis (NMA) is a type of meta-analysis that compares multiple treatments in a single analysis by combining the direct and indirect evidences (10). NMA overcomes the limitation of pairwise meta-analysis, which can only compare two interventions with direct comparison. Moreover, extension of comparison of the multiple treatment arms could identify the effectiveness ranking of the interventions. In previous studies, NMA was used to analyze the effect of different acupuncture therapies on neurological recovery in spinal cord injury (11) and chronic fatigue syndrome (12). However, comparisons of APSTs in treating patients with COPD are still insufficient.

Therefore, the aim of this systematic review and NMA is to analyze the effects on HRQL and FEV1% predicted of diverse APSTs in treating patients with COPD.

## Materials and Methods

### Search Strategy

Seven major electronic databases were searched, including four English databases (PubMed, Embase, Cochrane Central Register of Controlled Trials, Cumulative Index to Nursing and Allied Health Literature) and three Chinese databases (China National Knowledge Infrastructure, Wanfang Data, and Airiti Library), and identified articles published from initiation until September 2019 without language restriction.

The following keywords were used in literature search: “COPD” or “Chronic obstructive pulmonary disease” or “Chronic obstructive” or “Chronic bronchitis” or “Emphysema” or “Pulmonary emphysema” or “Lung emphysema” or “COAD” or “COBD” or “Chronic airflow limitation” or “Chronic airflow obstruction” or “Chronic airflow obstructive” or “Chronic obstructive lung disease” or “Chronic obstructive airway disease” or “Chronic obstructive respiratory disease” combined with “acupuncture,” “moxibustion,” “acupressure,” “electroacupuncture,” “Acu-TENS,” “ear acupuncture” in English databases. We also used Chinese synonyms of the keywords in Chinese databases.

### Study Selection Criteria

Only randomized controlled trials (RCTs) were included in this analysis. The target population comprised patients with COPD with stable condition. The diagnosis of COPD was based on the GOLD guidelines (3). The stable condition was defined as without acute exacerbations, increases of rescue medication, and unscheduled visits due to aggravating COPD for 3 months (3). All the retrieved studies comprised at least two comparative treatment arms, one arm comprising APST, which is in accordance with the World Health Organization definition of interventions (13), and the other arm comprising Tx (conventional medication or pulmonary rehabilitation) or control (without any treatment). The treatment definitions and abbreviations are listed in Table 1. We excluded studies with Chinese herbal medicine interventions or treatments without specific acupuncture point. The bibliographies of included review articles and clinical trials were manually reviewed for relevant references.

**Table 1:** Definitions of the acupuncture point stimulation treatments (APSTs).

**Abbreviation**	**Treatment**	**Definition**
A	Acupuncture	The insertion of needles into the body for medical purposes.
AA	Auricular acupuncture	Thin needles are inserted into specific points on the outer ear.
AB	Acupoint bloodletting	A superficial vein is pierced with a three-edged needle to let out a small amount of blood.
AE	Acupoint embedding	Embedding small needles into the skin at acupoints.
AM	Acupressure massage	Applying pressure at acupoints.
AST	Acupoint sticking therapy	Sticking the pasty medicinal extract preparations on acupoints.
AT	Acu-TENS	Transcutaneous electrical nerve stimulation at acupoints.
C	Control	Control or sham acupuncture.
EA	Electroacupuncture	Electric stimulation of the needle following insertion.
LA	Laser acupuncture	Laser irradiation at the acupoints.
Mx	Moxibustion	Burning ignited material (usually moxa) to apply heat at acupoints.
Tx	Conventional treatment	Conventional medication (M) or pulmonary rehabilitation (PR).
WA	Warm needle acupuncture	Burning moxa stick on the handle of the acupuncture needle following insertion.

### Data Extraction

We examined all the retrieved articles and extracted data using a preordained form. The following information was recorded: author, year, diagnosis, study design, sample number, intervention arms, patient characteristics, duration of COPD, SGRQ score, CAT score, and FEV1% predicted. The intervention details of APSTs were also recorded.

### Outcome Measurements

The primary outcome was HRQL measured by CAT or SGRQ before and after the intervention applied. CAT scores ranged from 0 to 40, and higher score indicates more limitations, whereas SGRQ scores ranged from 0 to 100, and higher score indicates a more severe impact of COPD on a patient's life. To analyze the improvement of HRQL together with SGRQ and CAT in NMA, we used standardized mean difference (SMD) from the baseline, and the value means that the HRQL improved by the intervention.

The secondary outcomes were FEV1% predicted, 6-min walk distance (6MWD), and risk ratio (RR) of the presence of adverse effects. FEV1% predicted is used to determine the severity of airflow limitation of patients with COPD (3). To analyze the improvement of FEV1% predicted, we used mean difference (MD) from the baseline, and the value means that the FEV1% predicted improved by the intervention.

### Risk of Bias Assessment

Two reviewers independently evaluated the methodological quality of the enrolled studies using the Cochrane RoB 2.0 tool (14). The assessment tool used five assessment domains for risk of bias to examine the quality of RCTs with overall bias. Discrepancies between the reviewers were solved through discussions with a third reviewer.

### Statistical Software

All the statistical analyses were performed using the network package in the statistical software STATA (version SE 14.0, StataCorp, College Station, TX, USA). We used R (version 3.6.1, R development Core Team) to generate figures. We also used Review Manager (RevMan) version 5.3 (Copenhagen: The Nordic Cochrane Centre, The Cochrane Collaboration, 2014) to assess the risk of bias summary and graph.

### Statistical Analyses

We performed random effect NMA using a consistency model. Each pair of interventions was compared by the SMD for HQRL and MD for FEV1% predicted. Ranking probabilities of each intervention were summarized by the 1,000 draws from the basic parameters in the network. The surface under the cumulative ranking curve (SUCRA) was calculated by the ranking probabilities for each intervention and used to determine the ranking. SUCRA is an index between 0 and 1, and the larger the SUCRA, the better the intervention.

### Publication Bias and Consistency Assessment

The publication bias was examined by using the funnel plot, and Egger's test was performed to assess the existence of small-study bias. Inconsistency assumption was assessed using three methods, including design-inconsistency, side-splitting model, and Lu and Ades inconsistency model.

## Results

### Study Identification

**Figure 1:**
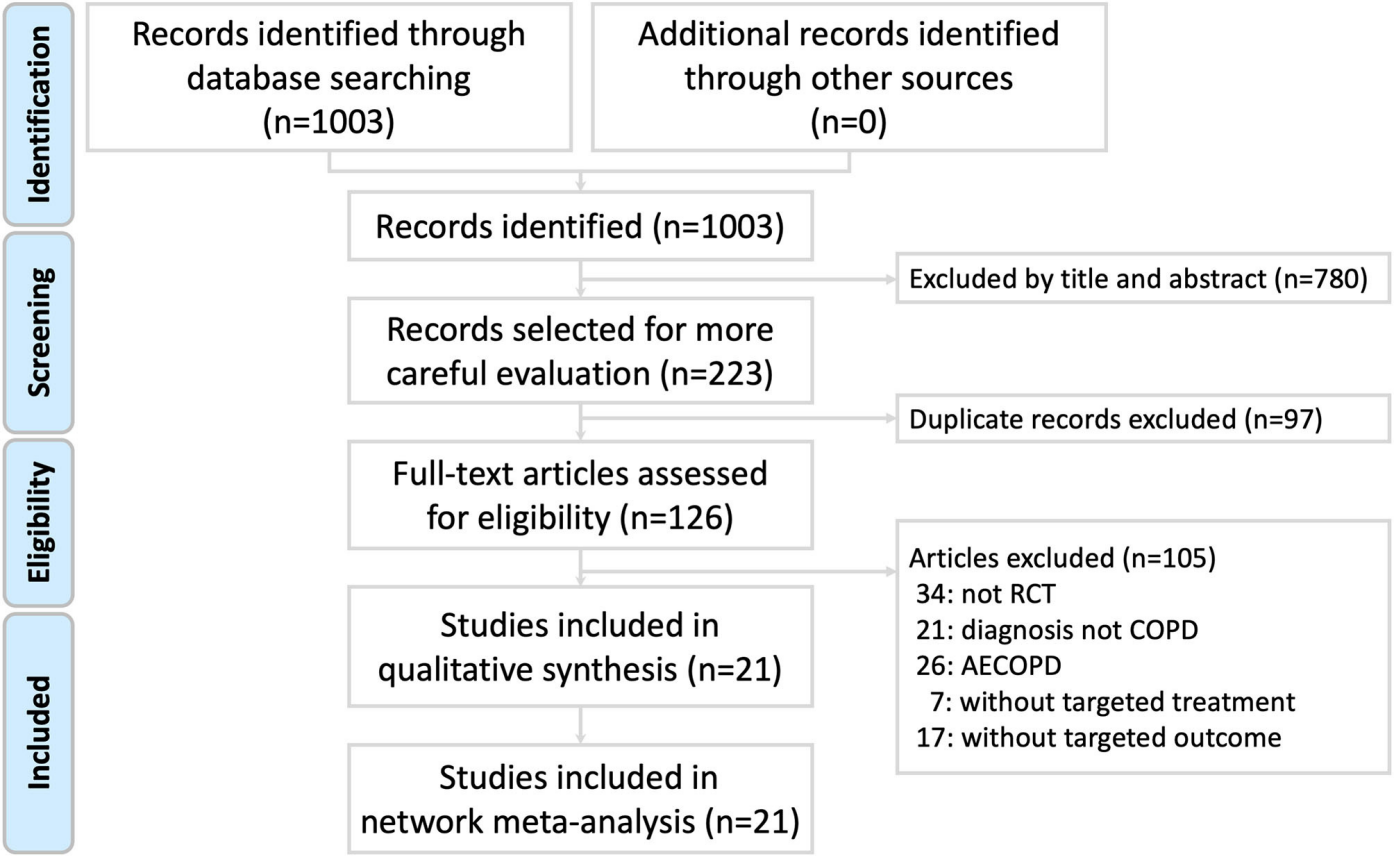
Preferred reporting items for systematic reviews and meta-analysis (PRISMA) study flow diagram.

The process was presented in a Preferred Reporting Items for Systematic Reviews and Meta-analysis (PRISMA) study flow diagram (Figure 1). A total of 1,003 studies were identified by the search terms. After eliminating the duplicate studies and excluding by reading the titles and abstracts, 126 articles were included for a full-text evaluation for eligibility. We excluded 105 studies (34 non-RCT studies, 21 studies whose diagnosis was not COPD, 26 studies that diagnosed acute exacerbation of COPD, 7 studies without target treatment, and 17 studies without targeted outcomes). Finally, 21 eligible studies were included into the risk of bias assessment and NMA.

### Characteristics of Included Patients and Treatments

The characteristics and study methodologies of the retrieved studies are shown in Table 2. The final quantitative analysis included 21 RCTs with 1,577 stable COPD participants with diverse severity GOLD stage II–IV (7, 15–34). Eighteen studies, which comprised 1,201 participants, assessing the participants' HRQL (SGRQ or CAT) by performing NMA. Seventeen studies, which comprised 1,114 participants, assessing the participants' FEV1% predicted by performing NMA. The baseline HRQL and lung function were well-balanced between the groups. The included studies had similar inclusion and exclusion criteria. Patients diagnosed with asthma or respiratory infection (in the weeks before enrollment) were excluded. All of the retrieved studies were parallel RCTs comparing the benefits of APSTs on HRQL or FEV1% predicted. The patients received the different duration of the treatments for 4 weeks to 2 years. Intervention details are presented in Supplementary Table 1.

**Table 2:** Summary of the retrieved studies.

**Author**	**Year**	**Study design**	**Double blind**	**Diagnosis**	**Intervention arms**		**Sample number (male/female)**	**Average age year, M(SD)**	**Duration of COPD year, M(SD)**	**Mean (SD) of baseline SGRQ**	**Mean (SD) of baseline CAT**	**Mean (SD) of baseline FEV1%**	**Overall bias (RoB 2.0)**	**Reference**
					**APSTs**	**Tx**								
Jie	2004	RCT parallel	No	COPD Stage II or III	A	M	22 (9/13)	61.0 (32.6)	11.7 (8.9)	–	–	70.9 (0.2)	High	(15)
					–	M + PR	22 (8/14)	60.0 (34.8)	12.1 (7.2)	–	–	71.1 (0.1)		
					A	M + PR	22 (10/12)	61.0 (33.2)	11.6 (9.0)	–	–	70.6 (0.7)		
Deering et al.	2011	RCT Parallel	No	COPD	A	PR	16 (8/8)	65.1 (9.7)	–	63.4 (10.5)	–	48.8 (22.7)	High	(16)
					–	PR	19 (12/7)	67.7 (5.3)	–	54.1 (14.1)	–	48.5 (16.1)		
					C	–	19 (12/7)	68.6 (5.5)	–	54.6 (14.1)	–	45.8 (18.3)		
Fan et al.	2011	RCT Parallel	No	COPD	WA	–	29 (13/16)	64.96 (8.71)	10.27 (5.71)	52.86 (7.28)	–	48.55 (5.05)	Some	(17)
					–	M	30 (12/18)	65.25 (10.68)	10.79 (5.26)	53.77 (6.74)	–	43.54 (6.29)		
Gao et al.	2011	RCT Parallel	No	COPD	WA	–	30 (13/17)	64.87 (8.73)	10.31 (5.82)	52.65 (6.77)	–	45.88 (5.05)	High	(18)
					–	M	30 (12/18)	65.25 (10.66)	10.78 (5.53)	53.06 (7.28)	–	43.54 (6.29)		
Suzuki et al.	2012	RCT Parallel	No	COPD	A	M	34 (31/3)	72.7 (6.8)	–	40.8 (15.4)	–	46.0 (16.6)	Low	(19)
					C	–	34 (32/2)	72.5 (7.4)	–	46.2 (14.2)	–	47.9 (16.5)		
Luo et al.	2013	RCT Parallel	No	COPD	Mx	PR	30 (20/10)	66.8 (6.9)	8.8 (3.5)	57.94 (7.64)	–	50.39 (2.71)	Some	(20)
					Mx	–	30 (19/11)	62.5 (5.6)	8.1 (2.3)	58.06 (1.35)	–	50.19 (2.85)		
					–	PR	30 (21/9)	65.1 (1.8)	8.4 (1.5)	57.48 (8.56)	–	50.14 (2.86)		
Xie et al.	2014	RCT Parallel	No	COPD	WA	–	40 (22/18)	68.9 (8.7)	11.8 (6.5)	–	–	45.89 (5.06)	Some	(21)
					–	M	40 (18/22)	68.5 (9.6)	12.3 (5.5)	–	–	43.55 (6.30)		
Yu	2014	RCT Parallel	No	COPD Stage II or III	WA	PR	30 (18/12)	63.0 (8.5)	8.9(3.7)	–	–	50.23 (2.56)	High	(22)
					–	M + PR	30 (17/13)	62.0 (7.6)	8.4 (3.5)	–	–	51.33 (2.43)		
Lee et al.	2015	RCT Parallel	No	COPD	WA	M	29 (19/10)	57.21 (6.68)	10.38 (4.9)	–	20.45 (4.37)	66.28 (6.86)	Low	(23)
					–	M	30 (17/13)	55.8 (7.23)	10.7 (4.88)	–	20.13 (5.30)	65.16 (6.16)		
Liu et al.	2015	RCT Parallel	No	COPD Stage III or IV	A	M	40 (24/16)	58.3 (12.4)	–	–	–	35.71 (7.28)	High	(24)
					–	M	40 (26/14)	63.2 (10.7)	–	–	–	36.42 (6.42)		
Liu X et al.	2015	RCT Parallel	No	COPD	AT	–	25 (10/15)	66.04 (8.815)	8.504 (7.11)	–	16.6 (5.8)	47.1 (19.2)	Low	(7)
					C	–	25 (15/10)	66.48 (9.368)	9.51 (9.93)	–	14.6 (6.0)	38.4 (18.7)		
Yang et al.	2016	RCT Parallel	No	COPD	WA	M	30 (19/11)	57.7 (8.41)	10.6 (5.06)	–	20.73 (6.42)	68.77 (13.83)	Low	(25)
					–	M	31 (18/13)	58.23 (7.77)	10.39 (5.12)	–	20.29 (4.80)	69.52 (13.30)		
Zang et al.	2016	RCT Parallel	No	COPD	AST	M	28 (16/12)	59.1 (11.6)	12.1 (1.3)	–	30.73 (7.92)	–	Some	(26)
					A + Mx + AST	M	32 (17/15)	57.4 (13.1)	11.9 (1.8)	–	31.21 (5.46)	–		
					–	M	36 (21/15)	58.7 (10.41)	11.7 (2.1)	–	31.06 (6.17)	–		
Deng et al.	2016	RCT Parallel	No	COPD	Mx	M + PR	100 (88/12)	63.1 (4.5)	7.42 (4.15)	45.73 (2.53)	–	–	Some	(27)
					–	M + PR	100 (86/14)	63.4 (7.3)	8.23 (1.32)	45.79 (2.49)	–	–		
Wang et al.	2017	RCT Parallel	No	COPD	A + Mx	M	50	–	–	54.12 (7.59)	–	–	High	(28)
					–	M	50	–	–	53.38 (9.12)	–	–		
Lee	2017	RCT Parallel	No	COPD	WA	PR	45 (27/18)	65.81 (2.75)	–	–	–	50.97 (2.37)	Some	(29)
					–	M + PR	45 (24/21)	62.74 (2.15)	–	–	–	51.04 (2.07)		
Shi et al.	2017	RCT Parallel	No	COPD	A	–	30 (18/12)	57.77 (6.54)	11.43 (4.37)	–	20.36 (4.20)	64.11 (5.79)	High	(30)
					–	M	31 (17/14)	55.9 (6.86)	11.68 (3.64)	–	20.23 (4.29)	65.85 (6.86)		
Nong et al.	2017	RCT Parallel	No	COPD	AM	M	54 (30/14)	68.39 (5.83)	6.46 (2.11)	–	30.37 (2.67)	–	Some	(31)
					–	M	54 (28/26)	69.11 (5.32)	6.39 (2.51)	–	30.76 (2.68)	–		
Tong et al.	2017	RCT Parallel	No	COPD	A	M + PR	22 (21/1)	65 (6)	9.1 (5.5)	–	13.50 (5.28)	40.76 (16.36)	Some	(32)
					–	M + PR	19 (15/4)	65 (7)	8.6 (6.8)	–	14.73 (5.37)	40.53 (17.40)		
Ge et al.	2017	RCT Parallel	No	COPD Stage II, III or IV	A	M + PR	22 (21/1)	65 (6)	9.1 (5.5)	–	–	40.76 (16.36)	Some	(33)
					–	M + PR	19 (15/4)	65 (7)	8.6 (6.8)	–	–	40.53 (17.40)		
Chen et al.	2018	RCT Parallel	No	COPD	EA	M	47 (30/17)	59.83 (5.16)	12.14 (3.16)	43.21 (4.56)	22.34 (2.25)	56.63 (3.15)	High	(34)
					–	M	47 (31/16)	60.29 (4.87)	11.98 (3.97)	43.59 (5.13)	22.47 (2.21)	55.97 (2.31)		

*A, acupuncture; AST, acupoint sticking therapy; AT, Acu-TENS; C, control (control or sham acupuncture); CAT, COPD assessment test; COPD, chronic obstructive pulmonary disease; EA, electroacupuncture; FEV1%, FEV1% predicted; M, conventional medication; Mx, moxibustion; PR, pulmonary rehabilitation; RCT, randomized controlled trial; SGRQ, st. george's respiratory questionnaire; Tx, conventional treatment (conventional medication or pulmonary rehabilitation); WA, warm acupuncture*.

### Risk of Bias Assessment

The risks of bias assessment of the retrieved studies are shown in Supplementary Figures 1A,B. The overall bias for each trial is also presented in Table 2. The quality was variable and showed a high concern in the “bias due to deviations from intended interventions” domain. None of the retrieved studies that uses a patient-blinded study design is the possible reason.

### Network Meta-Analysis

**Figure 2:**
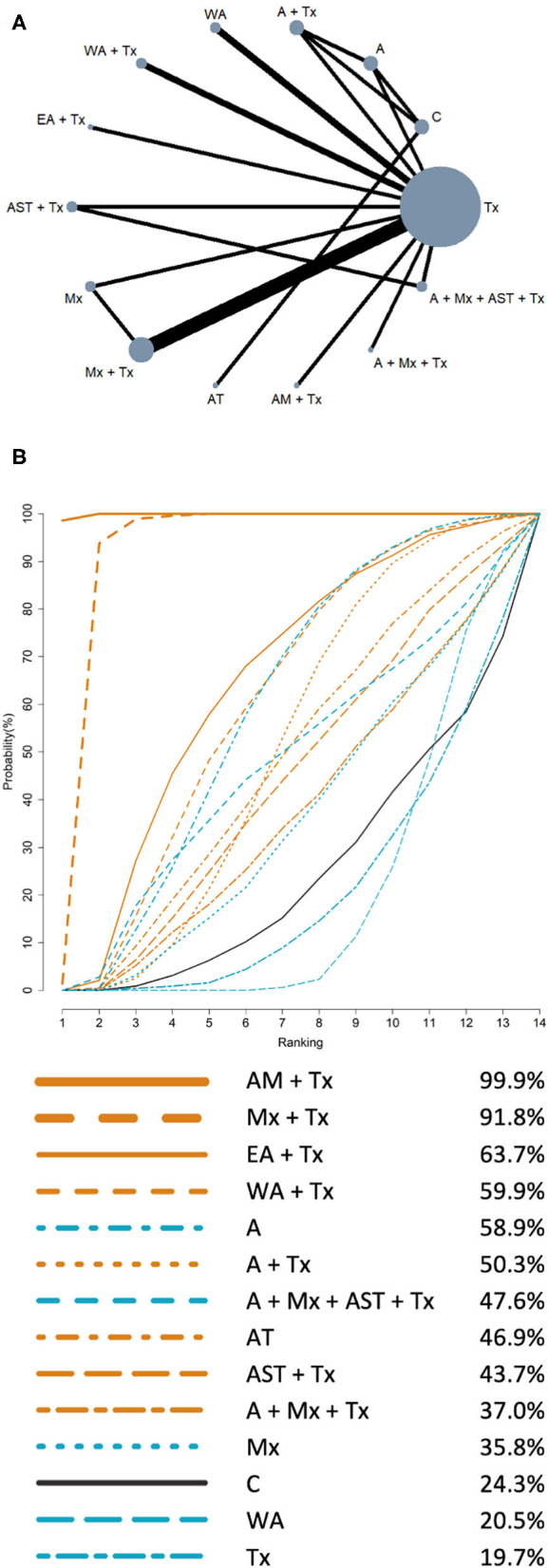
Network meta-analysis results of health-related quality of life: **(A)** Network plot; **(B)** SUCRA. **(A)** Network plot: the size of the nodes corresponds to the number of studies of each treatment. The lines between nodes represent direct comparison of the trials, and the thickness of the line linked between nodes corresponds to the number of trials included. **(B)** SUCRA: plot of the surface under the cumulative ranking curves. The larger the area under the curve means the higher the ranking. Ranking indicates the probability to be the best treatment (line color: orange: acupuncture point stimulation treatment combined with conventional treatment; blue: single intervention; black: control).

**Figure 3:**
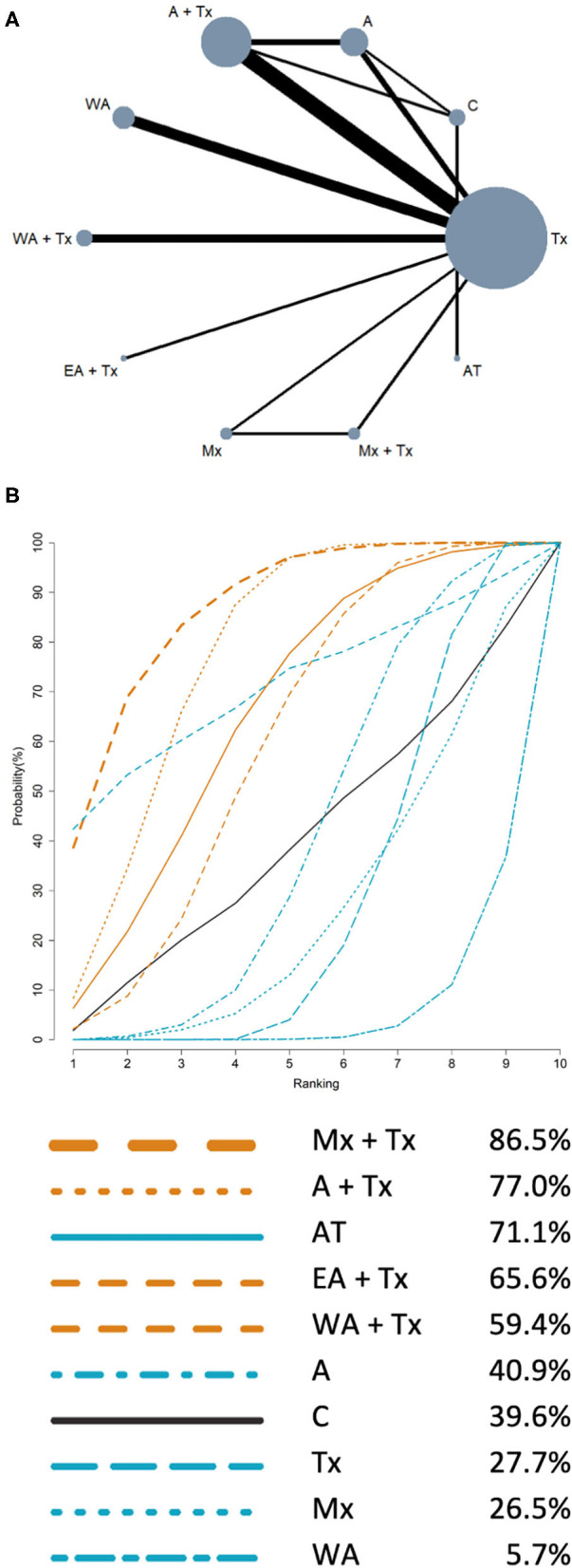
Network meta-analysis results of FEV_1_% predicted: **(A)** Network plot; **(B)** SUCRA. **(A)** Network plot: the size of the nodes corresponds to the number of studies of each treatment. The lines between nodes represent direct comparison of the trials, and the thickness of the line linked between nodes corresponds to the number of trials included. **(B)** SUCRA: plot of the surface under the cumulative ranking curves. The larger the area under the curve means the higher the ranking. Ranking indicates the probability to be the best treatment (line color: orange: acupuncture point stimulation treatment combined with conventional treatment; blue: single intervention; black: control).

The NMA results of HRQL and FEV1% are presented in Tables 3, 4, respectively. The summary of findings (network plot and SUCRA for HRQL and FEV1% predicted) is shown in Figures 2, 3, respectively.

**Table 3:** Network meta-analysis of health-related quality of life (HRQL).

**Control**	−0.62 (−2.68, 1.44)	−0.32 (−2.36, 1.73)	−5.09 (−7.25 to −2.92)^*^	−0.64 (−2.07, 0.78)	−2.84 (−4.65 to −1.02)^*^	−0.28 (−2.29, 1.73)	−0.54 (−2.61, 1.52)	−1.10 (−3.15, 0.95)	−0.93 (−2.75, 0.89)	0.11 (−1.71, 1.92)	−0.66 (−2.05, 0.73)	−0.92 (−2.29, 0.46)	0.03 (−1.49, 1.55)
0.62 (−1.44, 2.68)	**A** **+** **Mx** **+** **AST** **+** **Tx**	0.30 (−1.65, 2.25)	−4.47 (−6.54 to −2.39)^*^	−0.03 (−2.53, 2.48)	−2.22 (−3.93 to −0.50)^*^	0.34 (−1.58, 2.25)	0.08 (−1.32, 1.48)	−0.48 (−2.44, 1.47)	−0.31 (−2.03, 1.40)	0.73 (−0.98, 2.44)	−0.04 (−1.70, 1.62)	−0.30 (−2.08, 1.48)	0.65 (−0.75, 2.04)
0.32 (−1.73, 2.36)	−0.30 (−2.25, 1.65)	**A** **+** **Mx** **+** **Tx**	−4.77 (−6.83 to −2.71)^*^	−0.33 (−2.82, 2.16)	−2.52 (−4.21 to −0.83)^*^	0.04 (−1.86, 1.93)	−0.23 (−2.18, 1.73)	−0.78 (−2.72, 1.15)	−0.61 (−2.30, 1.08)	0.43 (−1.26, 2.11)	−0.34 (−1.98, 1.29)	−0.60 (−2.35, 1.15)	0.34 (−1.02, 1.71)
5.09 (2.92, 7.25)^*^	4.47 (2.39, 6.54)^*^	4.77 (2.71, 6.83)^*^	**AM** **+** **Tx**	4.44 (1.85, 7.03)^*^	2.25 (0.42, 4.09)^*^	4.81 (2.78, 6.83)^*^	4.55 (2.47, 6.63)^*^	3.99 (1.92, 6.05)^*^	4.16 (2.32, 5.99)^*^	5.20 (3.36, 7.03)^*^	4.43 (2.65, 6.21)^*^	4.17 (2.28, 6.06)^*^	5.11 (3.57, 6.65)^*^
0.64 (−0.78, 2.07)	0.03 (−2.48, 2.53)	0.33 (−2.16, 2.82)	−4.44 (−7.03 to −1.85)^*^	**AT**	−2.19 (−4.50, 0.12)	0.36 (−2.10, 2.83)	0.10 (−2.40, 2.61)	−0.46 (−2.95, 2.04)	−0.29 (−2.59, 2.02)	0.75 (−1.55, 3.06)	−0.01 (−2.00, 1.98)	−0.27 (−2.25, 1.71)	0.67 (−1.41, 2.75)
2.84 (1.02, 4.65)^*^	2.22 (0.50, 3.93)^*^	2.52 (0.83, 4.21)^*^	−2.25 (−4.09 to −0.42)^*^	2.19 (−0.12, 4.50)	**Mx** **+** **Tx**	2.56 (1.22, 3.89)	2.29 (0.58, 4.01)	1.74 (0.03, 3.44)	1.91 (0.49, 3.32)	2.95 (1.54, 4.35)	2.18 (0.83, 3.52)	1.92 (0.43, 3.41)	2.86 (1.86, 3.86)
0.28 (−1.73, 2.29)	−0.34 (−2.25, 1.58)	−0.04 (−1.93, 1.86)	−4.81 (−6.83 to −2.78)^*^	−0.36 (−2.83, 2.10)	−2.56 (−3.89 to −1.22)^*^	**Mx**	−0.26 (−2.18, 1.66)	−0.82 (−2.72, 1.08)	−0.65 (−2.30, 1.00)	0.39 (−1.26, 2.03)	−0.38 (−1.97, 1.21)	−0.64 (−2.35, 1.08)	0.31 (−1.01, 1.62)
0.54 (−1.52, 2.61)	−0.08 (−1.48, 2.18)	0.23 (−1.73, 2.18)	−4.55 (−6.63 to −2.47)^*^	−0.10 (−2.61, 2.40)	−2.29 (−4.01 to −0.58)^*^	0.26 (−1.66, 2.18)	**AST** **+** **Tx**	−0.56 (−2.52, 1.40)	−0.39 (−2.11, 1.33)	0.65 (−1.06, 2.36)	−0.12 (−1.78, 1.55)	−0.38 (−2.16, 1.40)	0.57 (−0.83, 1.97)
1.10 (−0.95, 3.15)	0.48 (−1.47, 2.44)	0.78 (−1.15, 2.72)	−3.99 (−6.05 to −1.92)^*^	0.46 (−2.04, 2.95)	−1.74 (−3.44 to −0.03)^*^	0.82 (−1.08, 2.72)	0.56 (−1.40, 2.52)	**EA** **+** **Tx**	0.17 (−1.53, 1.87)	1.21 (−0.49, 2.91)	0.44 (−1.20, 2.09)	0.18 (−1.58, 1.95)	1.13 (−0.25, 2.51)
0.93 (−0.89, 2.75)	0.31 (−1.40, 2.03)	0.61 (−1.08, 2.30)	−4.16 (−5.99 to −2.32)^*^	0.29 (−2.02, 2.59)	−1.91 (−3.32 to −0.49)^*^	0.65 (−1.00, 2.30)	0.39 (−1.33, 2.11)	−0.17 (−1.87, 1.53)	**WA** **+** **Tx**	1.04 (−0.37, 2.45)	0.27 (−1.07, 1.62)	0.01 (−1.47, 1.50)	0.96 (−0.04, 1.96)
−0.11 (−1.92, 1.71)	−0.73 (−2.44, 0.98)	−0.43 (−2.11, 1.26)	−5.20 (−7.03 to −3.36)^*^	−0.75 (−3.06, 1.55)	−2.95 (−4.35 to −1.54)^*^	−0.39 (−2.03, 1.26)	−0.65 (−2.36, 1.06)	−1.21 (−2.91, 0.49)	−1.04 (−2.45, 0.37)	**WA**	−0.77 (−2.11, 0.57)	−1.03 (−2.51, 0.46)	−0.08 (−1.07, 0.91)
0.66 (−0.73, 2.05)	0.04 (−1.62, 1.70)	0.34 (−1.29 1.98)	−4.43 (−6.21 to −2.65)^*^	0.01 (−1.98, 2.00)	−2.18 (−3.52 to −0.83)^*^	0.38 (−1.21, 1.97)	0.12 (−1.55, 1.78)	−0.44 (−2.09, 1.20)	−0.27 (−1.62, 1.07)	0.77 (−0.57, 2.11)	**A** **+** **Tx**	−0.26 (−1.37, 0.85)	0.69 (−0.21, 1.59)
0.92 (−0.46, 2.29)	0.30 (−1.48, 2.08)	0.60 (−1.15, 2.35)	−4.17 (−6.06 to −2.28)^*^	0.27 (−1.71, 2.25)	−1.92 (−3.41 to −0.34)^*^	0.64 (−1.08, 2.35)	0.38 (−1.40, 2.16)	−0.18 (−1.95, 1.58)	−0.01 (−1.50, 1.47)	1.03 (−0.46, 2.51)	0.26 (−0.85, 1.37)	**A**	0.95 (−0.16, 2.05)
−0.03 (−1.55, 1.49)	−0.65 (−2.04, 0.75)	−0.34 (−1.71, 1.02)	−5.11 (−6.65 to −3.57)^*^	−0.67 (−2.75, 1.41)	−2.86 (−3.86 to −1.86)^*^	−0.31 (−1.62, 1.01)	−0.57 (−1.97, 0.83)	−1.13 (−2.51, 0.25)	−0.96 (−1.96, 0.04)	0.08 (−0.91, 1.07)	−0.69 (−1.59, 0.21)	−0.95 (−2.05, 0.16)	**Tx**

*Estimates are presented as standardized mean difference (SMD) and 95% confidence interval. For the part in the upper right, the estimate of treatment effectiveness should be read as column-defining treatment compared to the row-defining treatment. An SMD of fewer than 0 favors the treatment in the column in improving HRQL. Blue grids indicate treatment in column better than treatment in row*.

* *Statistically significant*.

**Table 4:** Network meta-analysis of FEV_1_% predicted.

**Control**	5.90 (−5.74, 17.54)	6.98 (−5.12, 19.08)	−1.22 (−13.32, 10.88)	3.68 (−8.38, 15.73)	2.84 (−8.52, 14.21)	−4.46 (−15.59, 6.67)	4.98 (−5.72, 15.67)	0.52 (−9.94, 10.97)	−0.81 (−11.52, 9.90)
−5.90 (−17.54, 5.74)	**AT**	1.08 (−15.71, 17.86)	−7.12 (−23.91, 9.66)	−2.22 (−18.98, 14.53)	−3.06 (−19.32, 13.21)	−10.36 (−26.47, 5.75)	−0.92 (−16.73, 14.88)	−5.38 (−21.03, 10.26)	−6.71 (−22.53, 9.10)
−6.98 (−19.08, 5.12)	−1.08 (−17.86, 15.71)	**Mx** **+** **Tx**	−8.20 (−13.93 to −2.47)^*^	−3.30 (−11.20, 4.60)	−4.13 (−10.93, 2.67)	−11.44 (−17.84 to −5.03)^*^	−2.00 (−8.33, 4.33)	−6.46 (−13.23, 0.31)	−7.79 (−13.42 to −2.16)^*^
1.22 (−10.88, 13.32)	7.12 (−9.66, 23.91)	8.20 (2.47, 13.93)^*^	**Mx**	4.90 (−3.00, 12.80)	4.07 (−2.73, 10.87)	−3.24 (−9.64, 3.17)	6.20 (−0.13, 12.53)	1.74 (−5.03, 8.51)	0.41 (−5.22, 6.05)
−3.68 (−15.73, 8.38)	2.22 (−14.53, 18.98)	3.30 (−4.60, 11.20)	−4.90 (−12.80, 3.00)	**EA** **+** **Tx**	−0.83 (−7.55, 5.89)	−8.14 (−14.46 to −1.81)^*^	1.30 (−4.95, 7.55)	−3.16 (−9.85, 3.53)	−4.49 (−10.03, 1.05)
−2.84 (−14.21, 8.52)	3.06 (−13.21, 19.32)	4.13 (−2.67, 10.93)	−4.07 (−10.87, 2.73)	0.83 (−5.89, 7.55)	**WA** **+** **Tx**	−7.31 (−12.21 to −2.40)^*^	2.13 (−2.63, 6.90)	−2.33 (−7.70, 3.04)	−3.66 (−7.46, 0.15)
4.46 (−6.67, 15.59)	10.36 (−5.75, 26.47)	11.44 (5.03, 17.84)^*^	3.24 (−3.17. 9.64)	8.14 (1.81, 14.46)	7.31 (2.40, 12.21)	**WA**	9.44 (5.23, 13.65)	4.98 (0.14, 9.81)	3.65 (0.59, 6.70)
−4.98 (−15.67, 5.72)	0.92 (−14.88, 16.73)	2.00 (−4.33, 8.33)	−6.20 (−12.53, 0.13)	−1.30 (−7.55, 4.95)	−2.13 (−6.90, 2.63)	−9.44 (−13.65, 5.23)^*^	**A** **+** **Tx**	−4.46 (−8.52 to −0.40)^*^	−5.79 (−8.68 to −2.90)^*^
−0.52 (−10.97, 9.94)	5.38 (−10.26, 21.03)	6.46 (−0.31, 13.23)	−1.74 (−8.51, 5.03)	3.16 (−3.53, 9.85)	2.33 (−3.04, 7.70)	−4.98 (−9.81 to −0.14)^*^	4.46 (0.40, 8.52)	**A**	−1.33 (−5.09, 2.43)
0.81 (−0.90, 11.52)	6.71 (−9.10, 22.53)	7.79 (2.16, 13.42)	−0.41 (−6.05, 5.22)	4.49 (−1.05, 10.03)	3.66 (−0.15, 7.46)	−3.65 (−6.70 to −0.59)^*^	5.79 (2.90, 8.68)	1.33 (−2.43, 5.09)	**Tx**

*Estimates are presented as mean difference (MD) and 95% confidence interval. For the part in the upper right, the estimate of treatment effectiveness should be read as column-defining treatment compared to the row-defining treatment. An MD of more than 0 favors the treatment in the column in improving FEV1%. Blue grids indicate treatment in column better than treatment in row*.

* *Statistically significant*.

### Primary Outcome: Health-Related Quality of Life

For the outcome of HRQL, a total of 14 interventions and 3 three-arm trials, 12 two-arm trials were included. The network plot (Figure 2A) showed that the Tx group has the largest number of studies. The comparison between Tx and Mx + Tx has most trials for HRQL. Results of NMA for the treatment effectiveness of improving HRQL are presented in Table 3. In comparison with Tx, AM + Tx [−5.11; 95% confidence interval (CI), −6.65 to −3.57] was the most effective intervention in improving HRQL, followed by Mx + Tx [−2.86; 95% CI, −3.86 to −1.86]. The other treatments showed no significant difference. The rankings of treatment effectiveness in improving HRQL are presented in SUCRA (Figure 2B). Based on SUCRA, the best treatment in improving HRQL is AM + Tx (SUCRA = 99.9%) followed by Mx + Tx (91.8%), EA + Tx (63.7%), WA + Tx (59.9%), A (58.9%), A + Tx (50.3%), A + Mx + AST + Tx (47.6%), AT (46.9%), AST + Tx (43.7%), A + Mx + Tx (37.0%), Mx (35.8%), C (24.3%), WA (20.5%), and Tx (19.7%).

### Secondary Outcome: FEV1% Predicted

For the outcome of FEV1%, a total of 10 interventions and 3 three-arm trials, 15 two-arm trials were included. The network plot (Figure 3A) also showed that Tx has the largest number of studies. The comparison between Tx and A + Tx has the most trials. Results of NMA for the treatment effectiveness of improving FEV1% predicted are presented in Table 4. In comparison with Tx, Mx + Tx (7.79; 95% CI, 2.16 to 13.42) was the most effective in improving FEV1% predicted, followed by A + Tx (5.79; 95% CI, 2.90 to 8.68). The other treatments showed no significant difference with Tx. The rankings of treatment effectiveness in improving FEV1% predicted are presented in SUCRA (Figure 3B). Based on SUCRA, the best treatment in improving FEV1% predicted is Mx + Tx (86.5%), which is as similar as in improving HRQL, followed by A + Tx (77.0%), AT (71.7%), EA + Tx (65.6%), WA + Tx (59.4%), A (40.9%), C (39.6%), Tx (27.7%), Mx (26.5%), and WA (5.7%).

### Secondary Outcome: 6-min Walk Distance (6MWD)

Six included studies measured 6MWD as an exercise performance outcome (7, 19, 24, 28, 32, 33). The results demonstrated that APSTs significantly improves 6MWD. However, the studies only comprised A + Tx, AT, A + Mx + Tx, Tx, and C treatment arms. No further NMA was performed with insufficient arms.

### Secondary Outcome: Presence of Adverse Effects

None of the studies reported any adverse effects, and no further NMA was performed.

### Cluster Ranking Plot of Different Treatments

**Figure 4:**
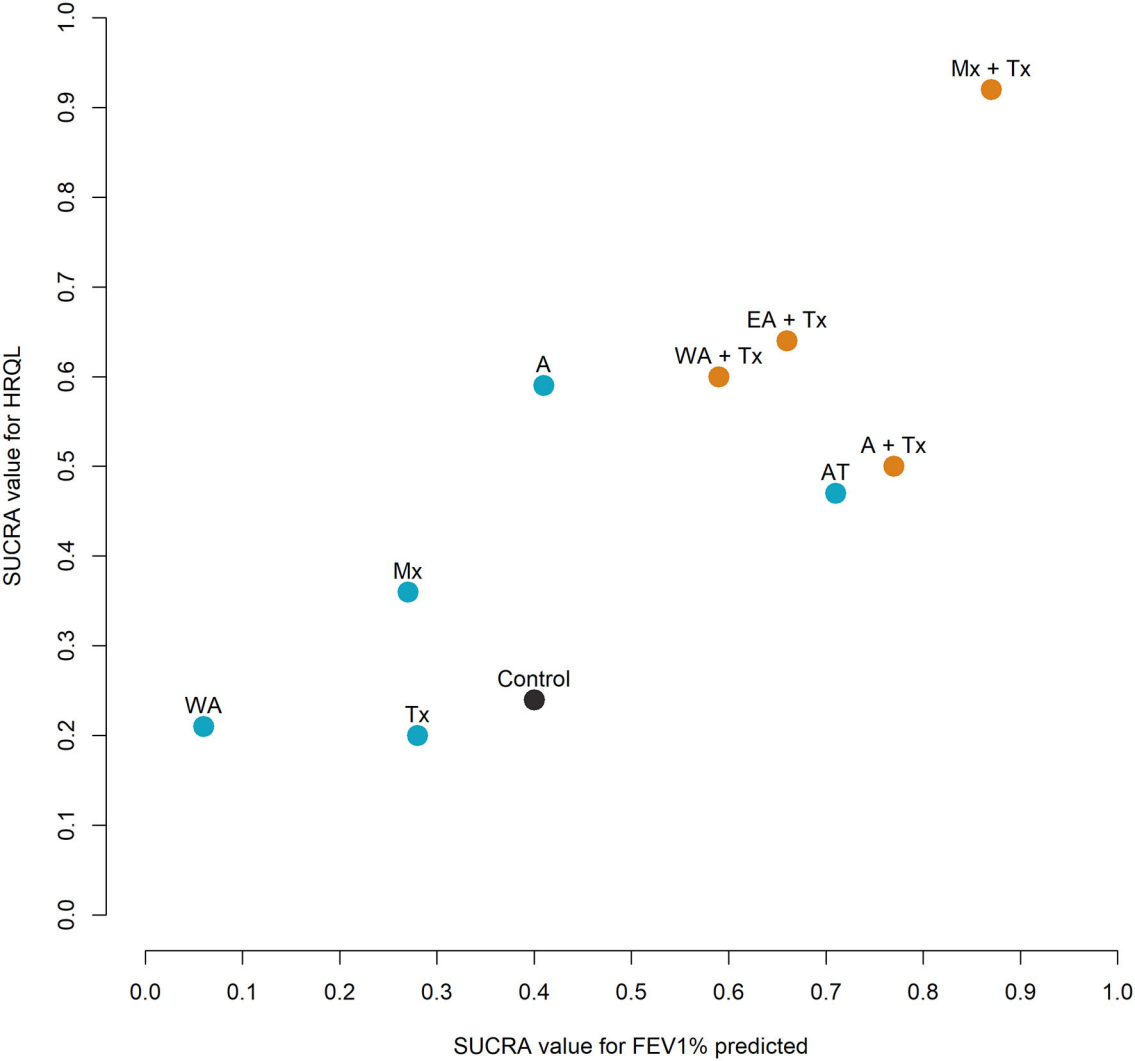
Clustered ranking plot. The plot is based on cluster analysis of surface under the cumulative ranking curves (SUCRA) values. Each plot shows the SUCRA values for two outcomes: health-related quality of life (HRQL) and FEV1%. Treatments lying in the upper right corner are more effective in HRQL and FEV1% than the other treatments (dot color: orange: acupuncture point stimulation treatment combined with conventional treatment; blue: single intervention; black: control).

The clustered ranking plot was based on cluster analysis of SUCRA values for HRQL and FEV1% predicted (Figure 4). Ten interventions with information of HRQL and FEV1% were analyzed. The results showed that compared to single intervention, combined intervention (APST + Tx) was more effective in improving both HRQL and FEV1% predicted.

### Publication Bias and Consistency Assessment

The results for assessing small-study bias, presenting the funnel plot for publication bias of HRQL and FEV1% predicted, are presented in Supplementary Figures 2, 3, respectively. There were no significant results of small-study bias, for both HRQL and FEV1% predicted. The consistency assessments of HRQL and FEV1% were presented in the Supplementary Figures 4, 5, respectively. There was no inconsistency detected for the treatment comparisons for both the outcome HRQL and FEV1% predicted.

## Discussion

This NMA, including 21 RCTs comprising 1,577 stable COPD participants, indicated that combined interventions (APSTs + Tx) are more effective than single intervention in improving both HRQL and FEV1% predicted. For patients with COPD, AM + Tx and Mx + Tx can significantly improve HRQL compared with Tx; Mx + Tx and A + Tx can significantly improve FEV1% predicted compared with Tx. We suggested that AM, Mx, and A can be considered effective non-pharmacological complementary interventions in treating patients with COPD.

The minimal clinically important difference (MCID) is a pivotal parameter that quantifies the threshold for clinically relevant change (35–37). It is reported that a decrease by 2.54 units in the CAT score meet the MCID (37). In the present study, we used SMD to analyze the improvement of HRQL with SGRQ and CAT together. In comparison with Tx, AM + Tx (−5.11 SMD; 95% CI, −6.65 to −3.57) was the most effective intervention in improving HRQL, followed by Mx + Tx (−2.86 SMD; 95% CI, −3.86 to −1.86). However, it is difficult to interpret the clinical relevance by results presented as SMDs directly (38). To elucidate the clinical meaning, we calculated an absolute difference in means by multiplying the SMDs by an estimate of the SD associated with CAT, suggested by the Cochrane Handbook for Systematic Reviews of Interventions (38). We conducted a conventional meta-analysis in the random-effects model by RevMan 5.4 (39) to estimate the SD associated with CAT by calculating a weighted average across all intervention groups of all the included studies that used CAT as a parameter (38) (Supplementary Figure 6). The obtained estimated SD associated with CAT was 2.62. The overall results demonstrated that the AM + Tx group improved 13.39 units in CAT score more than the Tx group; the Mx + Tx group improved 7.49 units in CAT score more than the Tx group, both greater than the estimated MCID for CAT (2.54 units). The results demonstrated better therapeutic effects in CAT with clinical relevance of AM + Tx and Mx + Tx compared to Tx. Regarding FEV1% predicted, it is reported that increased 4% meet the MCID (40). In the present study, the Mx + Tx group showed 7.79% (95% CI, 2.16 to 13.42) greater than the Tx group; the A + Tx group showed 5.79% (95% CI, 2.90 to 8.68) greater than the Tx group. The results demonstrated a better therapeutic effect in FEV1% predicted with clinical relevance of Mx + Tx and A + Tx compared to Tx.

Wang et al. suggested that acupuncture therapy may be effective and safe in improving HRQL and pulmonary function in patients with COPD (41). Hsieh et al. demonstrated that body acupuncture therapy is an effective and safe adjunctive treatment to improve HRQL in patients under optimal medical treatment (5). Fernández-Jané et al. indicated that in stable COPD patients, filiform needle acupuncture added to usual treatment improved dyspnea, HRQL, and exercise capacity (42). Fernández-Jané also suggested that acupressure could improve dyspnea, HRQL, and anxiety in patients with stable COPD (43). These meta-analysis studies revealed the effectiveness of acupuncture therapy, specifically A and AM, in treating patients with COPD. However, the studies were conventional pair-wise meta-analysis. This present study summarized different APSTs and identified the effectiveness ranking of the interventions by NMA. The results showed that AM, Mx, or A could be considered better clinical options among the diverse APSTs. Otherwise, in accordance with this present study, all of the studies above indicate the concern of evidence quality due to higher heterogeneity and lower methodological quality. Hence, the results should be interpreted prudently.

In treating patients with COPD by acupuncture, improvements of exercise tolerance (44), improvement of nutritional status (6), and respiratory muscle strength enhancement (6) were demonstrated to be possible mechanisms. Acupuncture improved peak oxygen uptake, peak minute ventilation, time to the limit of tolerance, and total SGRQ score of patients with COPD (44). Acupuncture also improved the nutritional state of patients with COPD, including body weight, respiratory muscle strength, nutritional hematological examination, and inflammatory biomarkers (6). According to the previous association rule analysis, ST36, BL12, and CV17 and ST36, BL12, and EXB1 could be considered the core acupoint combinations for the treatment of COPD (45).

Previous studies demonstrated that in patients with stable COPD, supplemented with AM to Tx or pulmonary rehabilitation programs can better relieve dyspnea and anxiety (46, 47), and improve respiratory rates, oxygen saturation (47), and HRQL (47, 48). AM can also improve perceived dyspnea and anxiety in patients with COPD using prolonged mechanical ventilation (49). The treatment periods range from 2 months to 2 years. The possible mechanisms of AM include decreased sympathetic stimulation to relaxation (49) and increased β-endorphin secretion to improve the effectiveness of breathing movements (50). AM is a noninvasive, self-administered, equipment-free intervention. However, the effect of AM highly depends on sustained and correct practice on acupoints, which leads to different outcomes. Additionally, more rigorous RCTs with a higher number of participants are still required. Therefore, we suggest that AM can be considered as a health instruction for patients with COPD.

According to the results, Mx is an effective treatment for COPD. Previous studies demonstrated that the mechanisms of Mx include thermal effects, radiation effects, and pharmacological actions of moxa and its combustion products (51). However, the mechanism directly related to treating patients with COPD is still unclear.

Despite undergoing optimal treatments suggested by the GOLD guidelines, many patients with COPD still experience dyspnea, impaired exercise capacity, fatigue, worse HRQL, and poor mental status (52). Even with emerging evidence in treating patients with COPD, there are still many challenges in clinical care. The selection of COPD therapy is often inconsistent with guidelines or evidence (53). Pulmonary rehabilitation (PR) is rarely a part of management in primary care (53). International estimates posit that only 1–2% of COPD patients receive PR because it is relatively inconvenient (54). GOLD guideline 2021 recommends that PR with supervised exercise training takes at least twice a week for 6 to 8 weeks to achieve optimum benefits (52). Lan et al. demonstrate that hospital-based PR program (training session with lower-limb cycle ergometer exercise for 40 min and monitored by a respiratory therapist, two sessions per week) develops significant improvements for at least eight sessions (55). COPD patients have to take more time and effort to complete effective PR programs compared to bronchodilators and oxygen therapy (54). The clinical challenges in treating patients with COPD may also relate to medical resources allocation, medical accessibility, and socioeconomic reasons (53). GOLD guideline 2021 suggests that acupuncture and acupressure as part of the non-pharmacological approaches in patients with advanced COPD that may improve breathlessness and HRQL (52). Moreover, acupuncture and other APSTs are more convenient and feasible for patient with lower cost and more accessible, especially in Asia. Consequently, it can be received regularly and frequently to achieve more benefits. Our results enhance the evidence of choosing AM, Mx, or A as complementary interventions in treating patients with COPD under conventional treatments and PR.

There are several limitations in this NMA. First, several trials provided insufficient information of study design, specifically the random sequence generation and allocation process, which led to risk of bias with some concerns. Second, the important clinical outcomes were presented roughly rather than comprehensively, such as the total score of SGRQ but without scores of different domains. We could only analyze and provide general results. Finally, these studies did not assess the effects in COPD with different severities respectively. It is still unclear whether APTSs are beneficial in patients with mild or severe COPD.

## Conclusions

Combined interventions (APSTs + Tx) are more effective than single intervention in improving both HRQL and FEV1% predicted. AM, Mx, and A can be considered effective non-pharmacological complementary interventions in treating patients with COPD under Tx.

## Data Availability Statement

The original contributions presented in the study are included in the article/Supplementary Material, further inquiries can be directed to the corresponding author.

## Author Contributions

C-LT, C-CL, and P-CH: conceptualization. Y-CW, I-ST, and P-CH: methodology. Y-CW and I-ST: software. Y-CW and P-CH: validation. C-LT, Y-CW, and P-CH: formal analysis and original draft. C-WW, C-YK, P-SH, and C-TL: investigation. P-CH and C-CL: review & editing. Y-CW: visualization. C-CL: supervision and funding acquisition. P-CH: project administration. All authors contributed to the article and approved the submitted version.

## Conflict of Interest

The authors declare that the research was conducted in the absence of any commercial or financial relationships that could be construed as a potential conflict of interest.
