# Supplementary material for: Acupuncture Point Stimulation Treatments Combined With Conventional Treatment in Chronic Obstructive Pulmonary Disease: A Systematic Review and Network Meta-Analysis
Source: Front Med (Lausanne). 2021 Jun 4;8:586900. doi: 10.3389/fmed.2021.586900 (PMC8211776; doi:10.3389/fmed.2021.586900)

Supplementary Material

**Supplementary Table 1. Summary of the intervention details**

| **Author** | **Year** | **Acupuncture point stimulation type** | **Intervention details** | **Acupuncture points** | **Treatment**  **period** | **APSTs treatment time (min.)** | **APSTs treatment**  **course** | **Ref.** |
| --- | --- | --- | --- | --- | --- | --- | --- | --- |
| Jia | 2004 | A | 2~3 Main Points + 1~2 Matching Points. Neutral supplementation and draining method with lifting-thrusting and twirling method till de qi, repeat once every 10 minutes during lasting needle. | Main points: BL13, BL43, LU9, KI3, BL23, ST36  Matching points: LU7, LU5, CV17, EX-B1 | Every other day for 100 days | 30 | 50 times for total | 15 |
|  |  | M | Conventional medication for COPD. |  | 100 days |  |  |  |
|  |  | PR | Endurance Exercise, Breathing Exercise. |  | 100 days |  |  |  |
| Deering et al | 2011 | A | Using European standard, sterile, single use needles in the direction and depth recommended. | LI11, LI10, TE10, TE6, L5, L7 | Once per week for 7 weeks | 20 | 7 times for total | 16 |
|  |  | PR | Pulmonary rehabilitation was undertaken for 2 hours, twice per week for 7 weeks, with a recommendation for 3 additional days of 30 minutes, unsupervised, home exercise programming. The first hour of each PR session consisted of a prescribed exercise training program based on the results of an Incremental Shuttle Walk Test. The second hour consisted of a multidisciplinary interactive educational session based on international guidelines. Patients also undertook inspiratory muscle training commencing at between 15% and 30% of maximum inspiratory pressure (PiMax) for 10 to 15 minutes per day, 5 days per week; progressing to a maximum of 60% of PiMax for 30 minutes (or 2-15 minutes). |  |  |  |  |  |
| Fan et al | 2011 | WA | 3 to 5 acupoints per time, manipulate till de qi, warming needle moxibustion was applied to the acupoints at back and ST36. The moxa sticks (10 mm in length) was inserted to the needle handle, ignite it and remove ashes when it burns out, two sticks for every point. | EX-B1, BL13, BL12, BL43, BL15, CV17, CV22, BL20, BL23, ST36 | Every other day, 3 times a week for 8 weeks | 30 | 24 times for total | 17 |
|  |  | M | Seretide (Salmeterol 50 μg + Fluticasone 250 μg), 1 puff per time. |  | 2 times a day for 8 weeks |  |  |  |
| Gao et al | 2011 | WA | Main points + 2~3 Matching Points. Warming needle moxibustion was applied to the acupoints at back and ST36. The moxa sticks (10 mm in length) was inserted to the needle handle, ignite it and remove ashes when it burns out, two sticks for every point. | Main points: EX-B1, BL13, ST36,  Matching points: BL12, GV14, BL43, BL15, CV17, CV22, BL20, BL23 | Every other day, 3 times weekly for 8 weeks, | 30 | 24 times for total | 18 |
|  |  | M | Seretide (Salmeterol 50 μg + Fluticasone 250 μg), 1 puff per time. |  | 2 times a day for 8 weeks |  |  |  |
| Suzuki et al | 2012 | A | Needles (0.35×70 mm, stainless steel; Dong Bang Acupuncture, Inc) were inserted to a depth ranging from 5 to 25 mm and were manually rotated clockwise and counterclockwise for 3 to 4 minutes at each point during a 50-minute treatment period. No electrical stimulation was performed. Perception of de qi (tingling, numbness, heaviness, and other feelings that occur after acupuncture needle insertion) during insertion and/or manipulation was confirmed at every point. | LU1, LU9, LI18, CV4, CV12, ST36, KI3, GB12 , BL13, BL20, BL23 | Once per week for 12 weeks. | - | 12 times for total | 19 |
|  |  | M | Conventional medication for COPD. |  | Daily for 12 weeks. |  |  |  |
| Luo et al | 2013 | Mx | Chinese herbs were grinded as a fine powder, and then the drug and ginger were blended in a certain proportion. The medicinal cakes having a length, a width and a height of 1×1×1 cm 3 respectively are prepared, and the texture of the medicinal cake is moderately wet and dry, and placed on a 4×5 cm 2 tape for use. | DU14, EX-B1, EX-HN15, BL13, BL23, BL20 | Once for a month, for 4 months | 240-480 |  | **20** |
|  |  | PR | Walking training with speed of 80 m/min. for 20-30 min. Breathing retraining including Diaphragmatic breathing twice a day and Pursed lip breathing 20 times a day. Muscle strength training for upper limbs with 0.6 kg weight dumbbell training twice a day. Endurance training for 30 min. once a day. |  | 4 months |  |  |  |
| Xie et al | 2014 | WA | Warming needle moxibustion was applied to the acupoints at back and ST36. The moxa sticks (10 mm in length) were inserted to the needle handle, ignite it and remove ashes when it burns out, two sticks for every point. | Main Points: ST36, BL13, EX-B1  Matching Points: BL43, BL15, GV14, BL12 | Every other day, 3 times a week for 8 weeks | 30 | 24 times for total | 21 |
|  |  | M | Seretide (Salmeterol 50 μg + Fluticasone 250 μg), 1 puff per time. |  | 2 times a day for 8 weeks |  |  |  |
| Yu | 2014 | WA | Warming needle moxibustion was applied to the acupoints. The moxa sticks (10 mm in length) were inserted to the needle handle in distance of 2-3 cm to skin, ignite it and remove ashes when it burns out, 3-4 sticks for every point. | Main Points: BL13, BL12, CV17, EX-B1, BL43, BL23, ST36  Matching Points: LU7, LU5, ST40, SP10 | Once a day, 5 times a week for 3 months | 3-4 times | 60 times for total | 22 |
|  |  | M | Conventional medication for COPD. |  | Once a day for 3 months |  |  |  |
|  |  | PR | Breathing retraining including Diaphragmatic breathing and Pursed lip breathing. Endurance training including Oxygen endurance training and Upper extremity exercise, once a day. |  | 3 months |  |  |  |
| Lee et al | 2015 | WA | Treatment started from Winter Solstice Festival (2014.7.18 to 2014.8.16). Warming needle moxibustion was applied to the acupoints. The moxa sticks (20 mm in length) were inserted to the needle handle, ignite it and remove ashes when it burns out, two sticks for every point. | GV14, BL13, BL20, BL23, BL17 | Every other day for 30 days | 30 | 15 times for total | 23 |
|  |  | M | Tiotropium Bromide Powder for inhalation, 1 puff (18 μg) per time. |  | Once a day for 30 days |  |  |  |
| Liu et al | 2015 | A | Disposable filiform needles of 0.25 mm in diameter and 40 mm in length were used to puncture patients in a supine lying position. Qihai (CV 6) and Guanyuan (CV 4) were punctured 1.0-1.3 cun perpendicularly, followed by even reinforcing-reducing manipulation upon arrival of qi. The needles were removed after 10 min. Then the patient was asked to sit and bend the head. Feishu (BL 13), Shenshu (BL 23), Dingchuan (EX-B 1), Danzhong (CV 17) and Zusanli (ST 36) were punctured. Feishu (BL 13) was punctured 0.5-0.8 cun obliquely toward the spine; Shenshu (BL 23) was punctured 0.5-1 cun perpendicularly; Dingchuan (EX-B 1) was punctured 0.5-1 cun obliquely toward the spine; Danzhong (CV 17) was punctured 0.5-0.8 cun subcutaneously; and Zusanli (ST 36) was punctured1-1.3 cun perpendicularly. Even reinforcing-reducing manipulation was applied upon arrival of qi. The needles were retained for 10 min. | BL13, BL23, CV6, CV4, EX-B1, CV17, ST36 | 2 times a week for 3 months | 20 | 24 times for total | 24 |
|  |  | M | Seretide inhaler, 50 μg/250 μg for each dose |  | 2 doses a day (once in the morning and once in the evening), for 3 months |  |  |  |
| Liu X et al | 2015 | AT | An acupuncturist wiped the skin of the points with a cotton ball soaked in 75% alcohol. Four pairs of electrodes for TENS (6805-D model, Shantou City Medical Equipment Co., Ltd., Shantou City, China) were placed on the acupoints and fixed with adhesive tape. Treatment frequency was set for 2Hz according to the study by Franco et al. | EX-B-1(Dingchuan), BL-13 (Feishu), BL-23 (Shenshu), ST- 36 (Zusanli) | Every 2 days for 4 weeks | 40 | 14 times for total | 7 |
| Yang et al | 2016 | WA | Treatment started from Winter Solstice Festival (2015.12.22 to 2016.1.17). Warming needle moxibustion was applied to the acupoints. The moxa sticks (20 mm in length) was inserted to the needle handle, ignite it and remove ashes when it burns out, two sticks for every points. | GV14, BL13, BL20, BL23, BL17 | Every other day for 27 days | 30 | 14 times for total | 25 |
|  |  | M | Tiotropium Bromide Powder for inhalation, 1 puff (18 μg) per time. |  | Once a day for 27 days |  |  |  |
| Zang et al | 2016 | AST | AST: Chinese herbs were grinded as a fine powder at a ratio of 1:3:1, and the texture is placed on a tape for use. | RN22, EX-B1 | Every 10 days for a month, for 6 months | - | 6 times for total | 26 |
|  |  | A | A:Take filiform needle to puncture the acupoints of LU5, LU7, ST40, RN17, KI3, ST36, leave the needle for 20min, once a day. | LU5, LU7, ST40, RN17, KI3, ST36 | Once a day, for 6 months | 20 |  |  |
|  |  | Mx | Mx: Use DAJ-4 moxibustion treatment instrument, take acupoints of lung acupoints BL13, one time moxibustion for 20 minutes, once a day. One month of treatment for 10 days is a course of treatment for 6 months. | BL13 | Once a day, every 10 days for a month as a session, for 6 months | 10 |  |  |
|  |  | M | Seretide inhaler, 50 μg/500 μg for each dose, twice a day. Tiotropium Bromide Powder for inhalation, 1 puff (18 μg) per time, once a day. |  | 6 months |  |  |  |
| Deng et al | 2016 | Mx | Chinese herbs were grinded as a fine powder, and then the drug and ginger were blended in a certain proportion. The medicinal cakes having a length, a width and a height of 1×1×0.5 cm 3 respectively are prepared, and the texture of the medicinal cake is placed on a tape for use. | DU14, EX-B1, BL13, BL43, DU9, BL23, RN22, RN17 | 6 times for a year, total 2 years | 240-360 | 12 times for total | 27 |
|  |  | M | Conventional medication for COPD. |  | 2 years |  |  |  |
|  |  | PR | Muscle strength training for upper limbs and lower limbs. Respiratory muscle strength training. Tai chi training for 30 min. once a day |  | 2 years |  |  |  |
| Wang et al | 2017 | A | Take filiform needle to puncture the acupoints of EX-B1, RN17, LU9, RN6, and use reinforcing action. | A: EX-B1, RN17, LU9, RN6 | Once a day, 7 times as a session, for 6 months | - | **-** | 28 |
|  |  | Mx | Take double-sided of BL43, EX-B18, BL13, BL23, three Zhuang (a dose unit of moxibustion) of moxa cone for each of the acupoint, | Mx: BL43, EX-B18, BL13, BL23 | Once a day, 7 times for 1 course. The treatment rested for 2 days for a total of 6 months. |  |  |  |
|  |  | M | Conventional medication for COPD. |  | 6 months |  |  |  |
| Lee | 2017 | WA | Warming needle moxibustion was applied to the acupoints. The moxa sticks were inserted to the needle handle. | BL13, BL12, CV17, EX-B1, BL43, BL23, ST36, LU5, ST40, SP10 | once a day, 3-5 times a week for 4 weeks | - | 12-20 times for total | 29 |
|  |  | M | Salbutamol Aerosol, 1 – 2 puff (100 – 200 μg) per time, less than 12 puff. |  | Once a day for 4 weeks |  |  |  |
|  |  | PR | Slow walking exercise for 60 minutes per day. |  | Once a day for 4 weeks |  |  |  |
| Shi et al | 2017 | A | Adopted the Shengsan Prescription for acupuncture. | Main Points: CV17, CV12, CV6, GV20, LI6, LU7  Matching Points: LU9, ST36 | 3 times a week for 2 months | 30 | 24 times for total | 30 |
|  |  | M | Tiotropium Bromide Powder for inhalation, 1 puff (18 μg) per time. |  | Once a day for 2 months |  |  |  |
| Nong et al | 2017 | AM | Let the patient rest quietly for 15 minutes and then take the prone position or sitting position. The operator first massages the patient's back with a massage for 2 minutes to relax the back muscles of the patient. The order of acupuncture points is from top to bottom, from left to right. First click on the acupuncture point on the acupuncture point for 1 min, then press for 2 min. Each acupoint is operated for 3 min, and 130-150 times per minute. The degree of suffering, the local acupuncture points are acid, swelling, numbness, pain or thermal sensation. After the end of the needle application, the skin around the acupuncture points is reddish and the skin temperature of the sputum is elevated. | BL13, BL18, BL25, BL20, BL23 | once a day, continuous for 2 weeks for a course of treatment, interval 2 weeks and then the next course of treatment, for 2 years | 24 | - | 31 |
|  |  | M | Conventional medication for COPD. |  |  |  |  |  |
| Tong et al | 2017 | A | The operator performs conventional acupuncture on the selected acupuncture points, leaving the needle for 30 min, and connected to the G6805-1A electroacupuncture instrument. The continuous wave is selected and the frequency is 1.7 Hz. The intensity and frequency can be adjusted for different patients. 2 to 3 times a week for a total of 14 treatments. | RN17, ST18, RN4, RN12, ST25, ST16 | 2-3 sessions a week | 30 | 14 sessions in total | 32 |
|  |  | M | Conventional medication for COPD. |  |  |  |  |  |
|  |  | PR | Aerobic training with exercise bike (JOHNSON B902) for total 40 min. 2 to 3 times a week for a total of 14 treatments. |  | 2-3 sessions a week |  | 14 sessions in total |  |
| Ge et al | 2017 | A | The operator performs conventional acupuncture on the selected acupuncture points, leaving the needle for 30 min, and connected to the G6805-1A electroacupuncture instrument. The continuous wave is selected and the frequency is 1.7 Hz. The intensity and frequency can be adjusted for different patients. 2 to 3 times a week for a total of 14 treatments. | RN17, ST18, RN4, RN12, ST25, ST16 | 2-3 sessions a week | 30 | 14 sessions in total | 33 |
|  |  | M | Conventional medication for COPD. |  |  |  |  |  |
|  |  | PR | Aerobic training with exercise bike (JOHNSON B902) for total 40 min. 2 to 3 times a week for a total of 14 treatments. |  | 2-3 sessions a week |  | 14 sessions in total |  |
| Chen et al | 2018 | EA | Regular needle insertion, after de Qi, the electroacupuncture treatment instrument (XYD-II type, Anyang Xiangyu Medical Equipment Co., Ltd.) conducts current stimulation, continuous wave, frequency 100 times/min, and needle retention for 30 min. Once every other day, 7 times for 1 course of treatment. | RN17, ST18, RN4, RN12, ST25 | Once for two days,7 times as a session, for 8 weeks | 30 | 28 times for total | 34 |
|  |  | M | Budesonide and Formoterol Fumarate Powder for Inhalation, one puff, twice a day |  | Twice a day for 8 weeks |  |  |  |

**Supplementary Figure 1. Risk of bias assessment (RoB 2.0)**

Review authors' judgement about each risk of bias item for each included study (RoB 2.0). (A) Risk of bias summary; (B) Risk of bias graph.


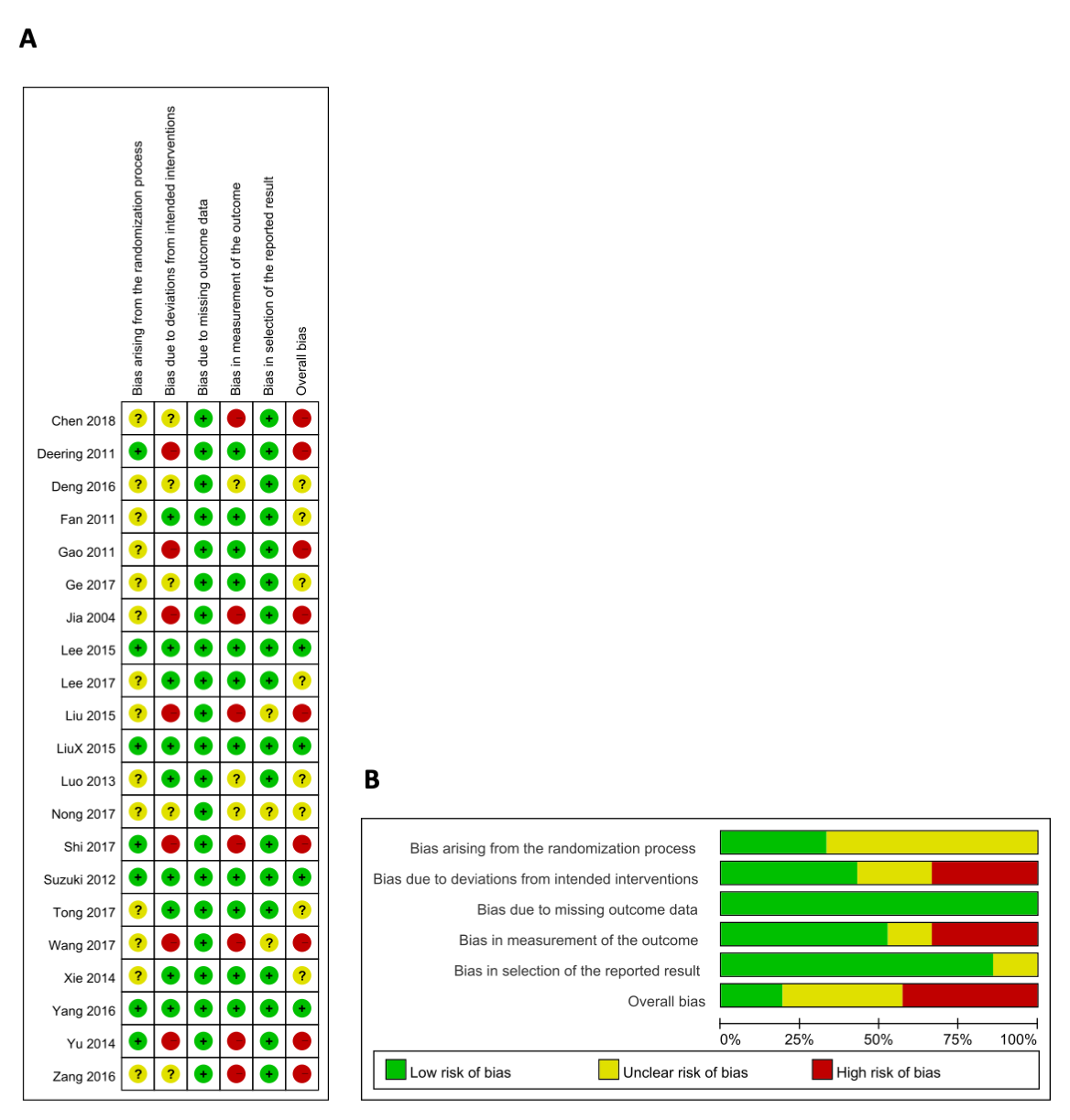


**Supplementary Figure 2. Publication bias of HRQL**

(A) Funnel plot; (B) Small study bias


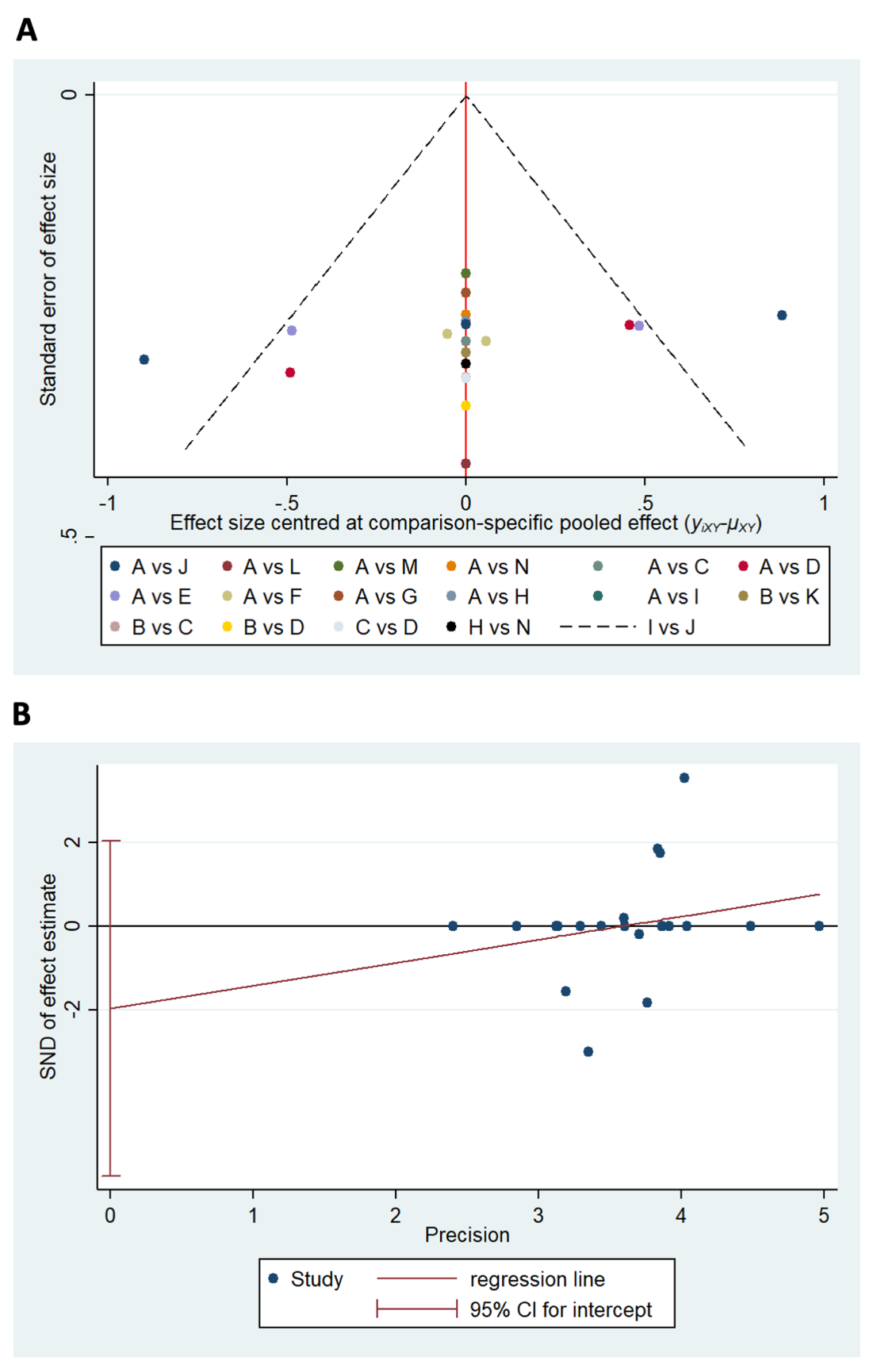


**Supplementary Figure 3. Publication bias of FEV_1_% predicted**

(A) Funnel plot; (B) Small study bias


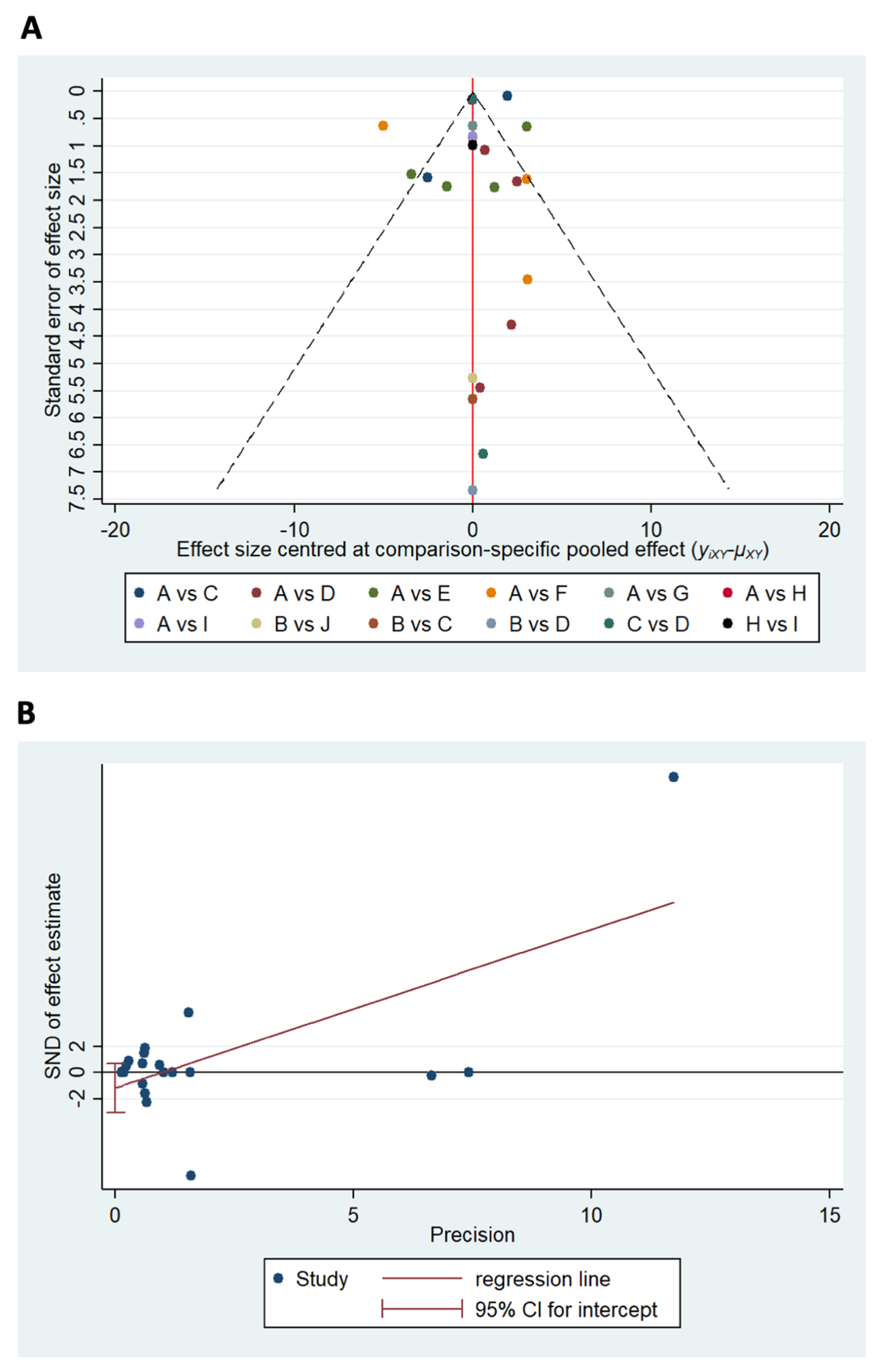


**Supplementary Figure 4. Consistency assessment of network meta-analysis of HRQL**

(A) Design inconsistency; (B) Side split; (C) Lu and Ades.

1. **Design inconsistency**

1. **Side split**

1. **Lu and Ades**

**Supplementary Figure 5. Consistency assessment of network meta-analysis of FEV_1_% predicted**

(A) Design inconsistency; (B) Side split; (C) Lu and Ades.

1. Design inconsistency

1. **Side split**

1. **Lu and Ades**

**Supplementary Figure 6**


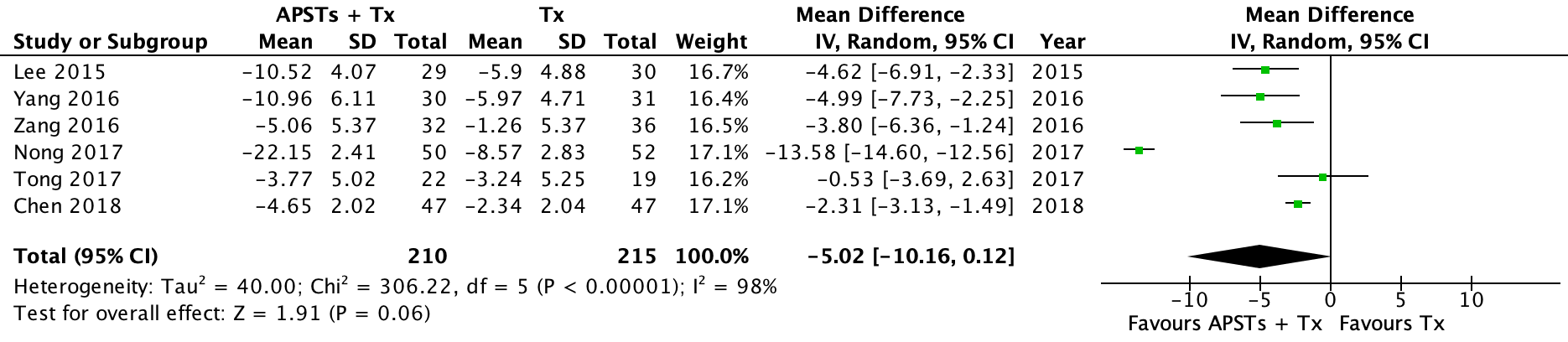

Supplement: Supplementary file 1 [file Data_Sheet_1.docx]
